# Phyto-ecological studies and distribution pattern of plant species and communities of Dhirkot, Azad Jammu and Kashmir, Pakistan

**DOI:** 10.1371/journal.pone.0257493

**Published:** 2021-10-06

**Authors:** Mevish Mumshad, Israr Ahmad, Shujaul Mulk Khan, Abdullah, Khadija Rehman, Mohammad Islam, Shazia Sakhi, Sami Ullah Khan, Sahib Gul Afridi, Sulaiman Shams, Samana Azam, Ishtiaq Ahmad, Rabia Afza, Zeeshan Ahmad

**Affiliations:** 1 Department of Botany, Women University of Azad Jammu & Kashmir, Bagh, Pakistan; 2 Department of Plant Sciences, Quaid-i-Azam University Islamabad, Islamabad, Pakistan; 3 Department of Biotechnology and Genetic Engineering, Hazara University Mansehra, Mansehra, Pakistan; 4 Center of Plant Sciences and Biodiversity, University of Swat, Mingora, Pakistan; 5 Department of Biochemistry, Abdul Wali Khan University, Mardan, Pakistan; 6 Department of Botany, Islamia College Peshawar, Peshawar, Pakistan; 7 Department of Botany, Hazara University Mansehra, Mansehra, KP, Pakistan; Mirpur University of Science and Technology, PAKISTAN

## Abstract

Plant species represent the hierarchical expression of vegetation as it is affected by various environmental gradients. We explored the plant species composition, distribution pattern, communities formation and their respective indicators under the influence of various environmental factors in the Dhirkot region, Azad Jammu and Kashmir. It was hypothesized that different environmental factors were responsible for the formation of various plant communities each with a distinct indicator. Quantitative ecological techniques were used for the sampling of vegetation. A total of 114 quadrats were established in 13 selected sampling sites. Phytosociological attributes were calculated for each plant species at each quadrat. Soil samples were collected and analyzed using different standard protocols. All the collected data were analyzed using Cluster Analysis, Indicator Species Analysis and Canonical Correspondence Analysis of PCORD and CANOCO software, respectively. A total of 145 plant species were recorded belong to 62 different families. Asteraceae and Lamiaceae were the dominant families, represented by 12 species each (8.27%). Cluster Analysis classify all the stations and plants into four major plant communities as 1) *Olea-Desmodium-Prunilla* community. 2) *Abies-Zanthoxylum-Pteracanthus* community 3) *Cedrus-Elaeagnus-Hypericum* community 4) *Alnus-Myrsine–Ranunculus* community. Soil pH, electrical conductivity, soil saturation, organic matter and altitude were the significant environmental factors that play its essential role in the plant species distribution, composition, formation of major plant communities and their respective indicators in the region. It is recommended that the identified indicator and rare plant species of the investigated area can further be grown for conservation and management purposes in *in-situ* environment.

## Introduction

Components of environmental gradients influence vegetation dynamics and their structure directly or indirectly [1,2]. The surrounding microclimatic conditions are responsible for general plant characters and traits, resulting in vegetation heterogeneity [3,4]. The vegetation of an area can be classified based on physiognomy and functional combination or on its component species that discriminate the physical appearance of vegetation. Species having structural and floristic traits with definite environmental tolerance are grouped into decipherable plant associations/communities. These plant associations are the mean of the largest biomes of the earth [5]. It gives us knowledge of how vegetation structure, habitat, habit, niche and species interaction of an ecosystem does exist [6]. Different factors like biotic and abiotic of an ecosystem are important phenomena for the ecological studies and investigations [7]. It affects plant population and vegetation heterogeneity as well. It changes vegetation origin and ecology in response to changes in these variables of a specific region. Various researchers have studied the interaction of environmental factors along with vegetation structure and composition [8,9]. These factors help to recognize the composition, diversity and distribution pattern of plants in different plant communities. Furthermore, topography (slope, aspect and elevation) affect the climate, temperature and evapotranspiration which in turn result in vegetation diversity [10]. Plant growth is also determined by edaphic factors which influenced by climate, topography, time, organism and parent materials [11]. Moreover, various scientists have used quantitative analysis in a different field. These measures revise vegetation features to show their effect on vegetation dynamics and associated flora.

The classification of various plants into different communities is essential to study natural resource management, habitat deprivation, and fragmentation [12]. The main principle of a quantitative study of vegetation is to explain the vegetation pattern and categorize it in a meaningful method. It also determines the species range, which shows individuals’ distribution among the communities in a specific environment [13]. Many qualitative, quantitative, and synthetic characteristics of plant communities, e.g., density, diversity, dominance, floristic composition, structure development, physical appearance, mutual exchange relations of plants, the environmental variables, and the classification of plant communities, are analyzed in the field of plant ecology. But the computer-based statistical and different analytical programs are very rarely used by the ecologist. It helps them analyze the influence of different environmental variables on species groups and discover structure in the data set [14,15]. These statistical programs overcome the complex multifold data by classifying vegetation and comparing the results to abiotic components [16–18]. It minimizes the complex structure of vegetation in a very simple way.

Ecologists in the past have used the concept of dominant species in the classification of vegetation into potential plant communities and association. Recently ecologist used multivariate statistics to provide more objective and unbiased classification of vegetation. The area under consideration in the current manuscript; the Dhirkot region of District Bagh, Azad Kashmir, has not been studied earlier via using robust multivariate statistical techniques. It was hypothesized that different environmental factors were responsible for the formation of various plant communities each with a distinct indicator. Is it possible to identify plant communities through indicator species approach in relation to environmental factors? Can this approach to vegetation classification be of help in conservation management? Therefore, this research was aimed to evaluate the plant communities’ formation, driving factors and indicator species of each group using the robust unbiased statistical approaches to explain the complex plant distribution patterns species composition and the underlying mechanism.

## Materials and methods

### Study area

**Fig 1:**
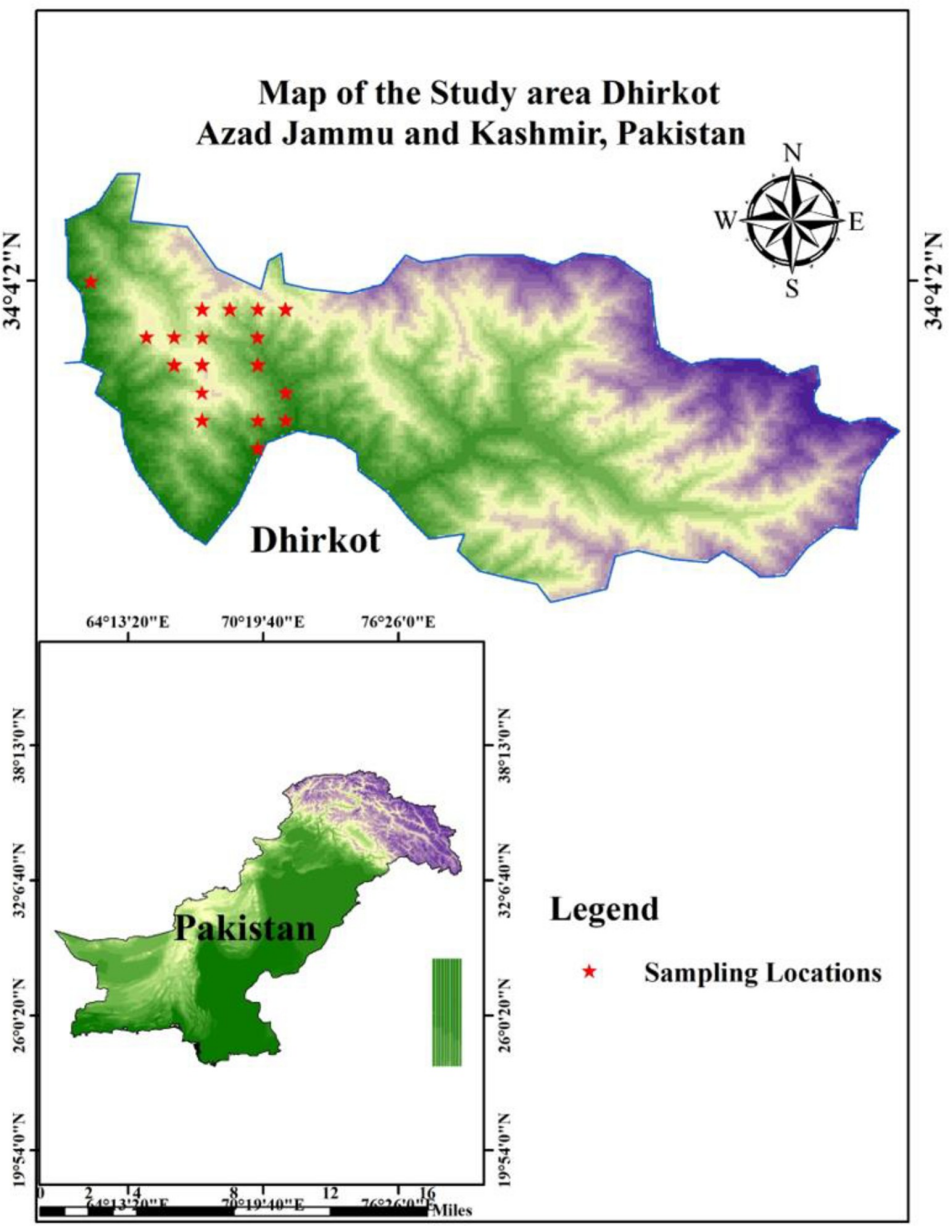
Map of study area Dhirkot Azad Jammu and Kashmir, Pakistan.

The Dhirkot region of Azad Jammu and Kashmir is located at 55km southeast of Muzaffarabad at 33° – 57° North latitude and 73° – 36° East longitude. It covers an area of 150 km^2^ with an elevation range of 600-2000m above sea level with moist temperate climate [19]. The area receives a significant amount of rainfall throughout the year. Maximum precipitation occurs in July i.e., 96mm while minimum precipitation occurs in November i.e., 16mm. The minimum temperature drops down to 4°C in winter while in summer high temperature reaches up to 24°C. Dhirkot region lies in the upper limit of the subtropical humid moist temperate pine zone, mainly comprising a deciduous mixed moderate coniferous forest. Most of the area is covered with plant species that includes *Pinus wallichiana* A.B.Jacks (Blue Pine), *Pinus roxburghii* Sarg (Chir Pine), with a variety of mosses, grasses, herbs and shrubs. The map of the study area is shown in (Fig 1).

### Vegetation sampling

A total of 13 altitudinal transects (with 114 quadrats) were established for the sampling of vegetation randomly (Fig 1). The sampling began at lowest elevation to the mountain peaks (600–2000 m.s.l.) using GPS (Global Positioning System) [20,21]. At each transect, 7–10 quadrats were taken at 100m intervals along with an elevation gradient. The size of the quadrats for trees, shrubs, and herbs were kept 100m, 25m, and 1m, respectively [22]. Data attributes i.e., density, frequency, cover, relative density, relative frequency, relative cover and Importance Value Index (IVI) were measured at each transect for each plant species [23–27]. The diameters of trees were measured at breast height (DBH) for the determination of its cover. These phytosociological attributes were assessed for the identification of indicators, plant species composition, distribution pattern, dominant and rare plants examination. The plant specimens were collected, marked with proper tags, and pressed with a plant presser during the field. Specimens were mounted on standard herbarium sheets having a size of (17.5 × 11.5 inch). All plant specimens were identified with the help of Tropicos New Flora of Pakistan (http://legacy.tropicos.org/Project/Pakistan), The Plant List (http://www.theplantlist.org/) and other expert taxonomists [28–31].

### Soil analyses

The soil samples were collected up to 15cm depth from each quadrat in 3 replicates, tagged, air-dried, and sieved to remove large particles [32–34]. The soil physicochemical properties i.e., soil pH, electrical conductivity (EC), total dissolved solids (TDS), soil saturation and organic matter concentration, were measured. Soil sample in grams was mixed with distill water at 1:5, shaken at 300 rpm and left the sample for 30 minutes each till the paste is formed. Then, the soil EC, pH and TDS were measured through EC, pH and TDS meters, respectively [35,36]. The organic matter was measured using Walky method [37]. According to it, one gram of soil sample was taken in 500 ml conical flask along with 10 ml Sulfuric acid (H_2_SO_4_) and Potassium dichromate (K_2_Cr_2_O_7_), shaken and placed the sample for 30 minutes to cool. After that 100–1500 mL distilled water, sodium fluoride (0.5g)/phosphoric acid (3mL) and 10 drops of indicator were added to the solution [38]. The organic matter was analyzed in soil samples using below formulae.

Organicmatterpercentage=6.67(T/S),

where T is the amount of iron sulfate (FeSO4) and S is the blank reading through the procedure of [37].

### Statistical analysis

All the collected data were analyzed to determine the relationship and impact of measured environmental variables on the vegetation of the region. The data of all transects (quadrats) and plants were sorted in MS EXCEL for Cluster Analysis according to the software requirements [39]. Indicator Species Analysis was carried out for the identification of indicator species using IVI data through PCORD software. A threshold level of 95% significance (p≤0.05) along with 20% indicator value was kept as cutoff for indicator species identification [16,40]. These identified indicators were used for the naming of plant communities. The species and environmental data were analyzed via CCA in CANOCO software version 4.5 to find the effect of environmental variables on species composition and distribution pattern.

## Results

A total of 145 plant species belonged to 62 families were recorded from Dhirkot region, District Bagh Azad Kashmir, Pakistan (S1 Table). Among these, 96 were herbs (66% of the total vegetation), 30 trees (21%) and 19 shrub species (13%). Lamiaceae and Asteraceae were recorded as the most dominant families, followed by Rosaceae (6.89%), Leguminaceae (6.20%) and Polygonaceae (5.51%). *Pinus roxburghii*, *Pinus wallichiana*, *Diospyros lotus*, *Berberis lycium*, *Dodonaea viscosa*, *Punica granatum*, *Avena fatua*, *Malvastrum coromandelianum* and *Impatiens edgeworthii* were the dominant plant species of the area. At the same time, *Alnus nitida*, *Malus* domestica, *Prunus armeniaca*, *Citrus x aurantium*, *Jasminum grandiflorum*, *Debregeasia salicifolia*, *Nerium oleander*, *Polygonum amplexicaulis*, *Fragaria vesca* and *Persicaria capitata* were the rare species of the studied area based on IVI.

### Result of cluster analysis

**Fig 2:**
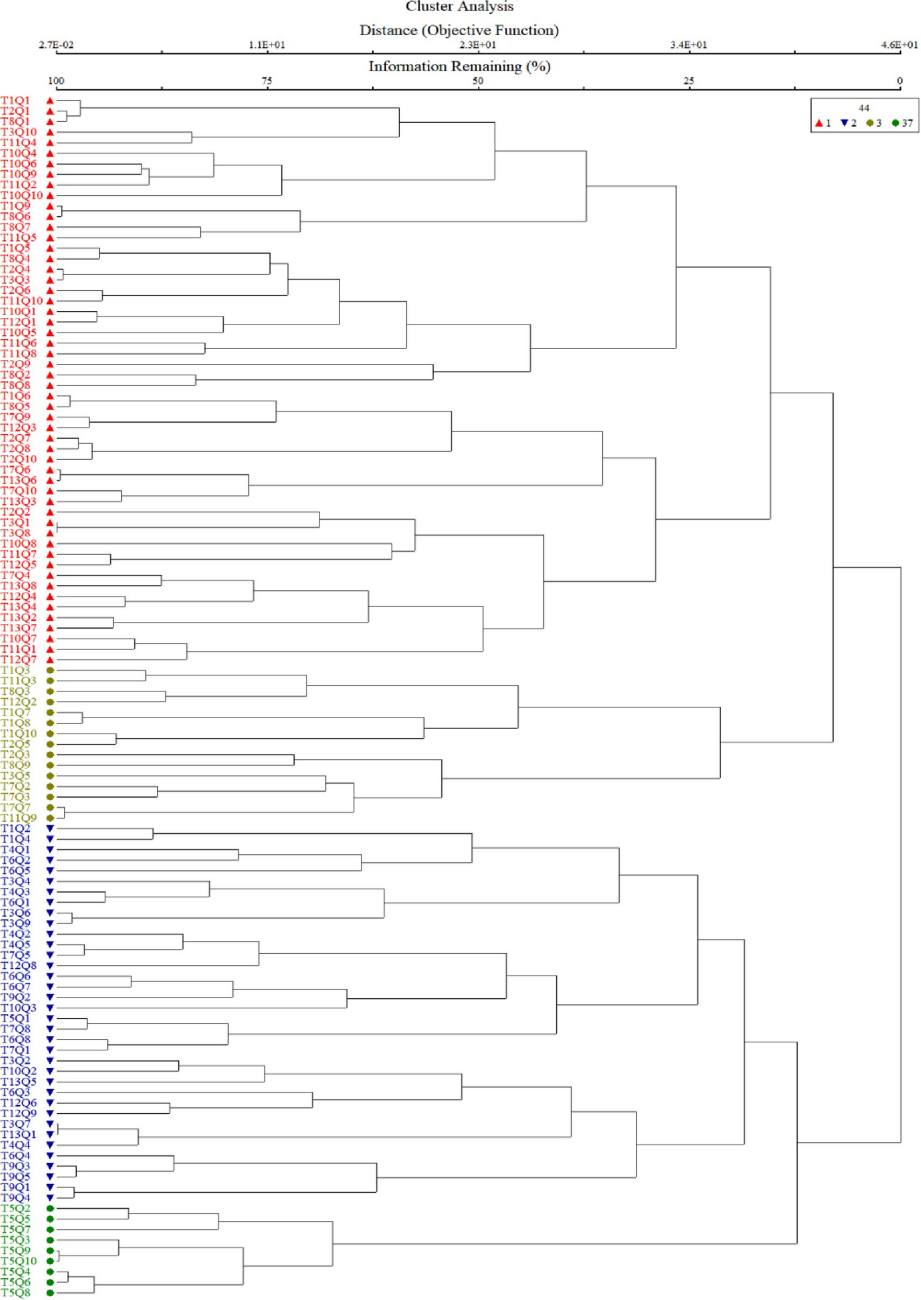
Cluster dendrogram showing communities classification based on Sorenson Distance Measurements and Ward Linkage Method of the region.

All the recorded plant species and quadrats were classified into four potential plant communities based on Sorenson Distance Measurements and Wards Linkage Method using PCORD software (Fig 2).

### Community classification

#### 1. *Olea–Desmodium—Prunella* plants community

**Fig 3:**
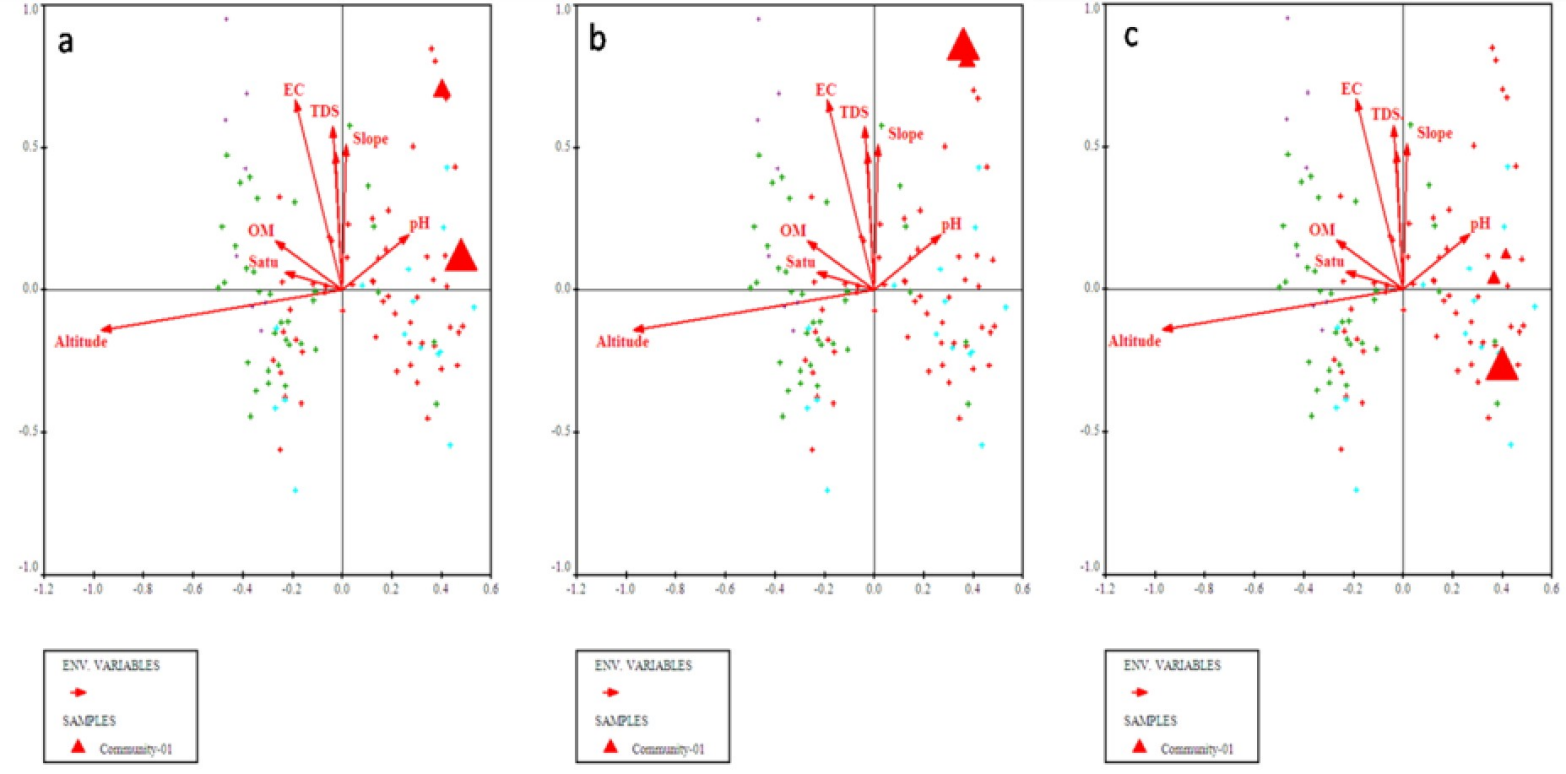
Bi-plots of (a) *Olea ferruginea*, (b) *Desmodium elegans*, and (c) *Prunella vulgaris* indicators along with measured environmental variables.

The community name was given based on Indicator Species Analysis. This community consists of 54 different quadrats/stations and 112 different plant species (25 trees, 16 shrubs and 71 herb species), at elevation range from 968-1699m. The topmost indicator species of this community were *Oleo ferruginea*, *Desmodium elegans* and *Prunella vulgaris* based on Indicator species value greater than 20% and Probability less than 0.05 (Fig 3). The dominant trees of this community were *Pinus roxburghii*, *Bauhinia variegata*, *Diospyros lotus*, *Aesculus indica*, *Ficus palmata* and rare tree species were *Ailanthus altissima*, *Prunus armeniaca*, *Morus nigra*, *Pyrus communis* and *Prunus persica* based on IVI values. The dominant shrubs revealed *Dodonaea viscosa*, *Punica granatum*, *Indigofera heterantha* and *Berberis lycium* with higher IVI value. Simultaneously, rare shrubs included *Elaeagnus umbellata*, *Debregeasia salicifolia*, *Machilus odoratissima* and *Nerium oleander* with low IVI in the region. The dominant herbs included *Avena fatua*, *Malvastrum coromandelianum*, *Rumex dentatus*, *Campanula pallida*, *Pteris cretica*, *Mentha longifolia* and *Arthraxon prionodes* was the rare herb species of this plant community. The soil state pf this community significantly varied, and it could be one of the factor for distinctive plant indicators as compared to others. This community has pH between 6.2–8.62, electrical conductivity varies from 7.4–62 ppm, TDS 6.35–305 ppm, organic matter 0.34–2.46% and soil saturation between 37–43%.

#### *Abies-Zanthoxylum-Pteracanthus* plants community

**Fig 4:**
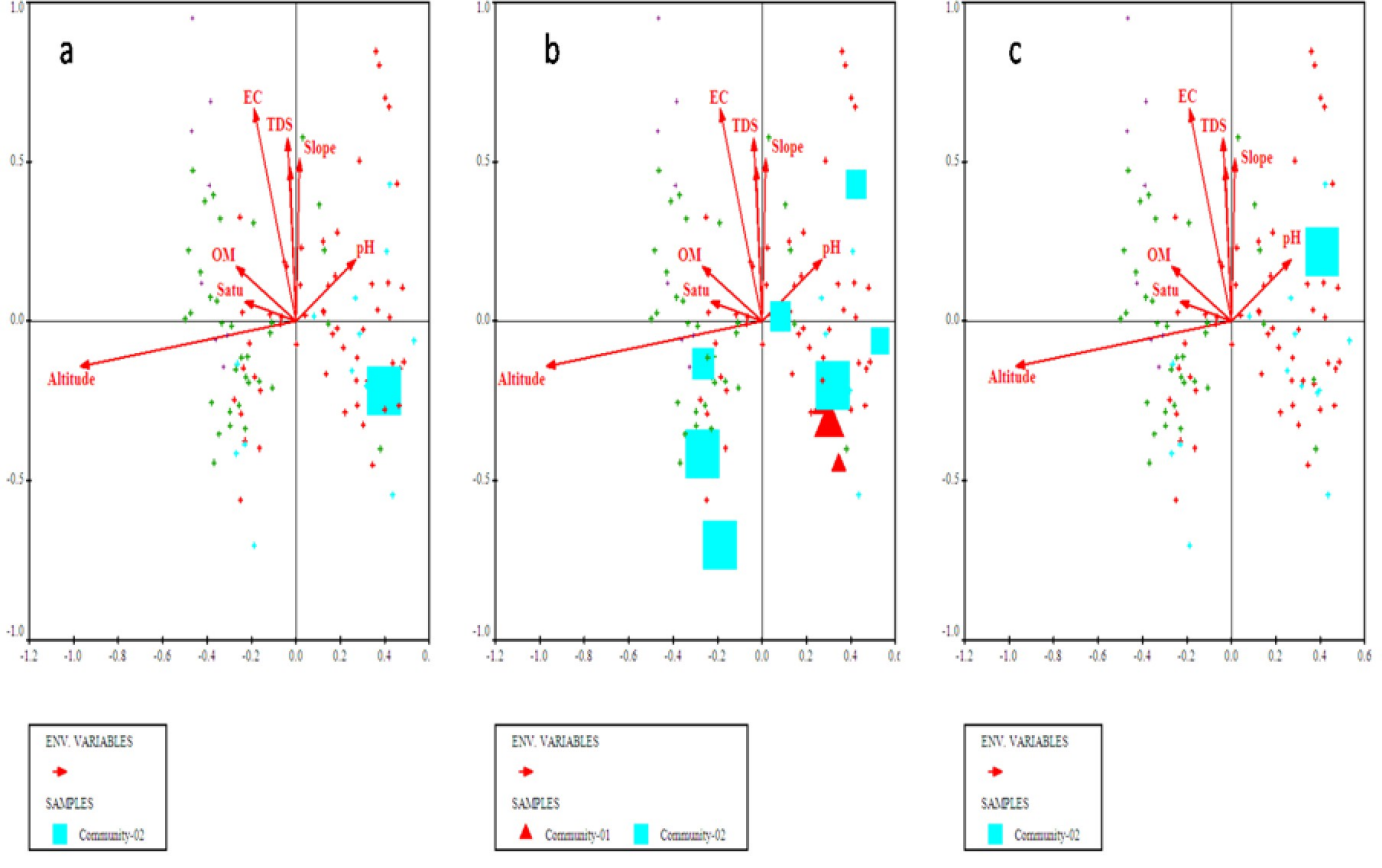
Bi-plot of *Abies pindrow*, *Zanthoxylum alatum* and *Pteracanthus urticifolius* with prevailing environmental variables.

This community consisted of 15 different quadrats/stations and 43 different plant species (12 trees, 9 shrubs and 22 herbs) at an elevation range of 972-1696m. The topmost indicator species of this community were *Abies pindrow*, *Zanthoxylum alatum* and *Pteracanthus urticifolius* based on probability (≤0.05) and indicator values (>20%) (Fig 4). The dominant trees of this community were *Eucalyptus alba*, *Pinus roxburghii* and *Morus nigra* while rare tree species included *Citrus × aurantium*, *Morus alba* and *Ailanthus altissima*. The dominant shrubs revealed *Punica granatum*, *Berberis lycium* and rare shrub species including *Ricinus communis* and *Jasminum grandiflorum*. The recorded dominant herbs were *Cannabis sativa*, *Malvastrum coromandelianum*, *Impatiens edgeworthii*, *Fragaria nubicola* and *Plantago lanceolata*. At the same time, *Boenninghausenia albiflora*, *Scutellaria chamaedrifolia*, *Hydrocotyle asiatica*, *Hedera nepalensis* and *Polygonum amplexicaulis* were the rare herb species of this plants community. This community has a pH range between 6.8–8.7, electrical conductivity 12.9–59 ppm, TDS 14–74 ppm, organic matter 0.51–2.25% and soil saturation between 35–43%.

#### *Cedrus- Elaeagnus -Hypericum* plants community

**Fig 5:**
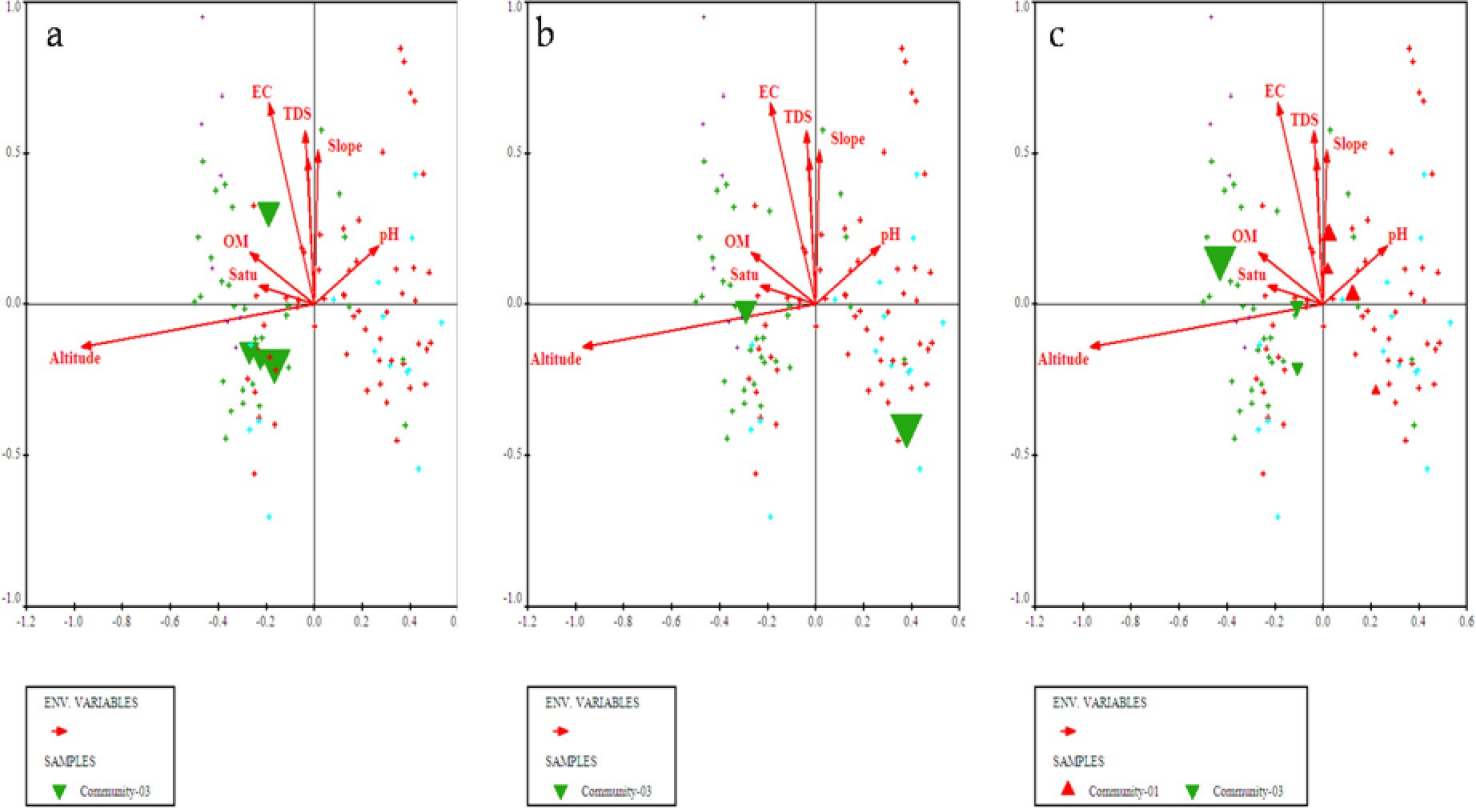
CCA bi-plot of top most indicators i.e., (a) *Cedrus deodara*, (b) *Elaeagnus umbellata* and (c) *Hypericum perforatum* concerning measured environmental factors.

This community included 36 different stations/quadrats and 92 different plant species (23 trees, 17 Shrubs and 52 herbs) at an elevation range of 1097-1800m. The topmost indicator species of this community were *Cedrus deodara*, *Elaeagnus umbellata* and *Hypericum perforatum* (Fig 5). Dominant trees of this community were *Pinus wallichiana*, *Quercus incana*, *Pinus roxburghii* and *Diospyros lotus*, while rare tree species consisted of *Acaccia arabica*, *Eucalyptus alba*, *Platanus orientalis* and *Morus alba*. The dominant shrubs revealed *Berberis lyceum* and *Machilus odoratissima* with high IVI and rare shrubs included *Debregeasia salicifoli*a, *Dodonaea viscosa* and *Jasminum grandiflorum* with low IVI in the region. The herbaceous layer was dominated by *Dryopteris filix*, *Fragaria nubicola*, *Impatiens edsgeworthii*, *Campanula pallida*, *Pteris cretica* and *Euphorbia helioscopia*. While *Trifolium pretense*, *Parthenium hysterophorus*, *Silybum marianum*, *Viola* canescens, *Ajuga parviflora* were the rare herb species of this community. Soil pH ranged from 5.91 to 8.29, electrical conductivity 8.19 to 83 ppm, TDS 10 to 109 ppm, organic matter 0.7 to 2.56 and soil saturation ranged between 35 to 43%.

**Fig 6:**
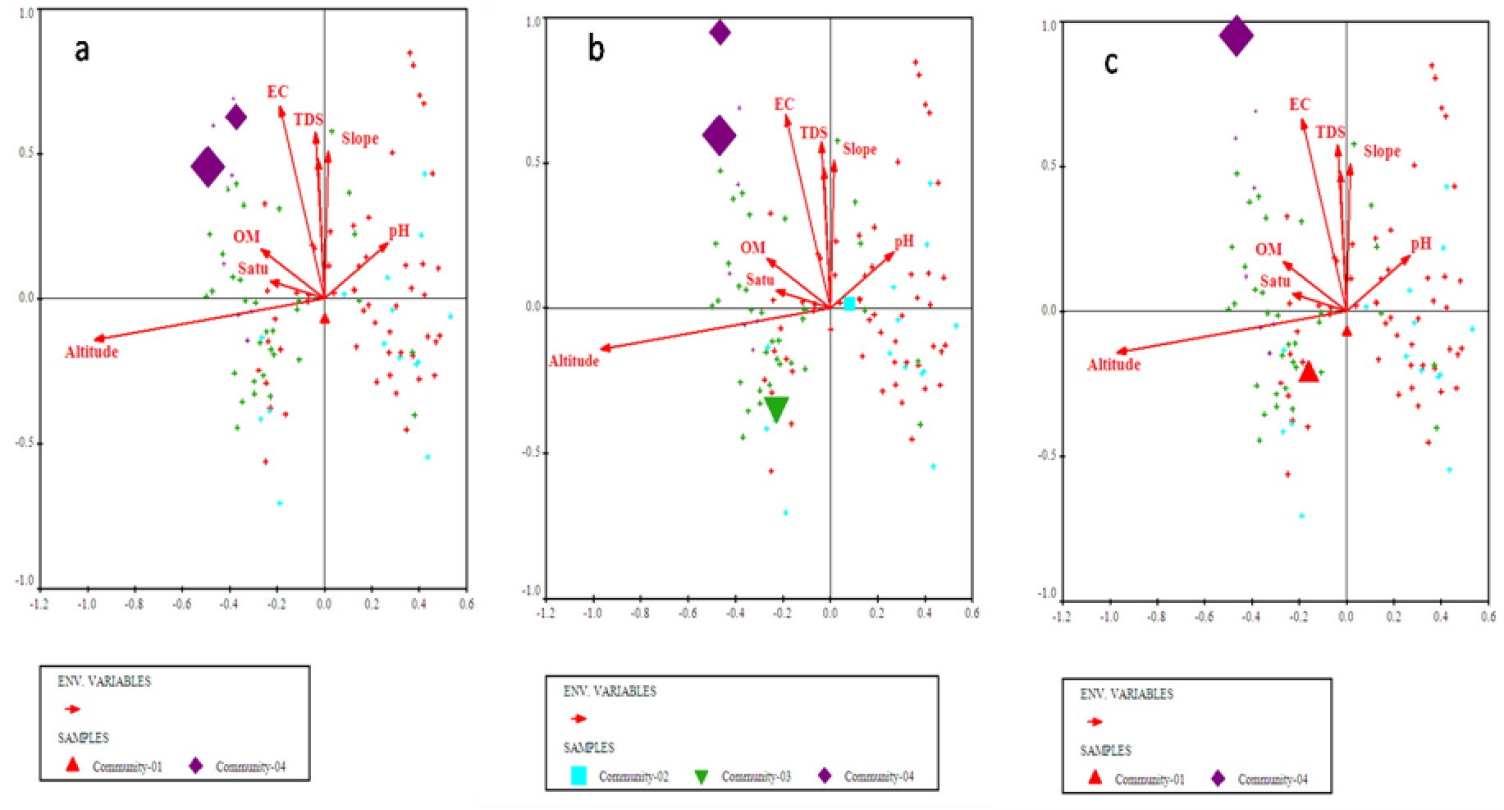
Bi-plot of *Alnus nitida*, *Myrsine africana* and *Ranunculus arvensis* with measured environmental variables after CCA of CANOCO software.

*Alnus–Myrsine- Ranunculus* plants community. This community consisted of 9 different quadrats/stations and 44 different plant species (9 trees, 17 shrubs and 18 herbs) at an elevation range of 1681–1738 m. The topmost indicator species of this community were *Alnus nitida*, *Myrsine africana* and *Ranunculus arvensis* based on indicator values greater than 20% and probability values less than 0.05 (Fig 6). Dominant trees of this community were *Pinus wallichiana* and *Morus nigra* rare tree species consisted of *Melia azedarach* and *Bauhinia variegata*. The dominant shrub included *Indigofera heterantha*, *Elaeagnus umbellata* and rare shrubs were *Wikstroemia canescens* and *Debregeasia salicifolia*. The dominant herbs were *Hedera nepalensis* and *Cymbopogon martini*, *Silybum marianum* were rare herb species of this plant community. Soil pH of this community ranged between 6.9–7.9, electrical conductivity varied from 22–83.4 ppm, TDS 32–112, organic matter 0.82 to 2.43% and soil saturation ranged between 40–43%.

### Environmental gradient

**Fig 7:**
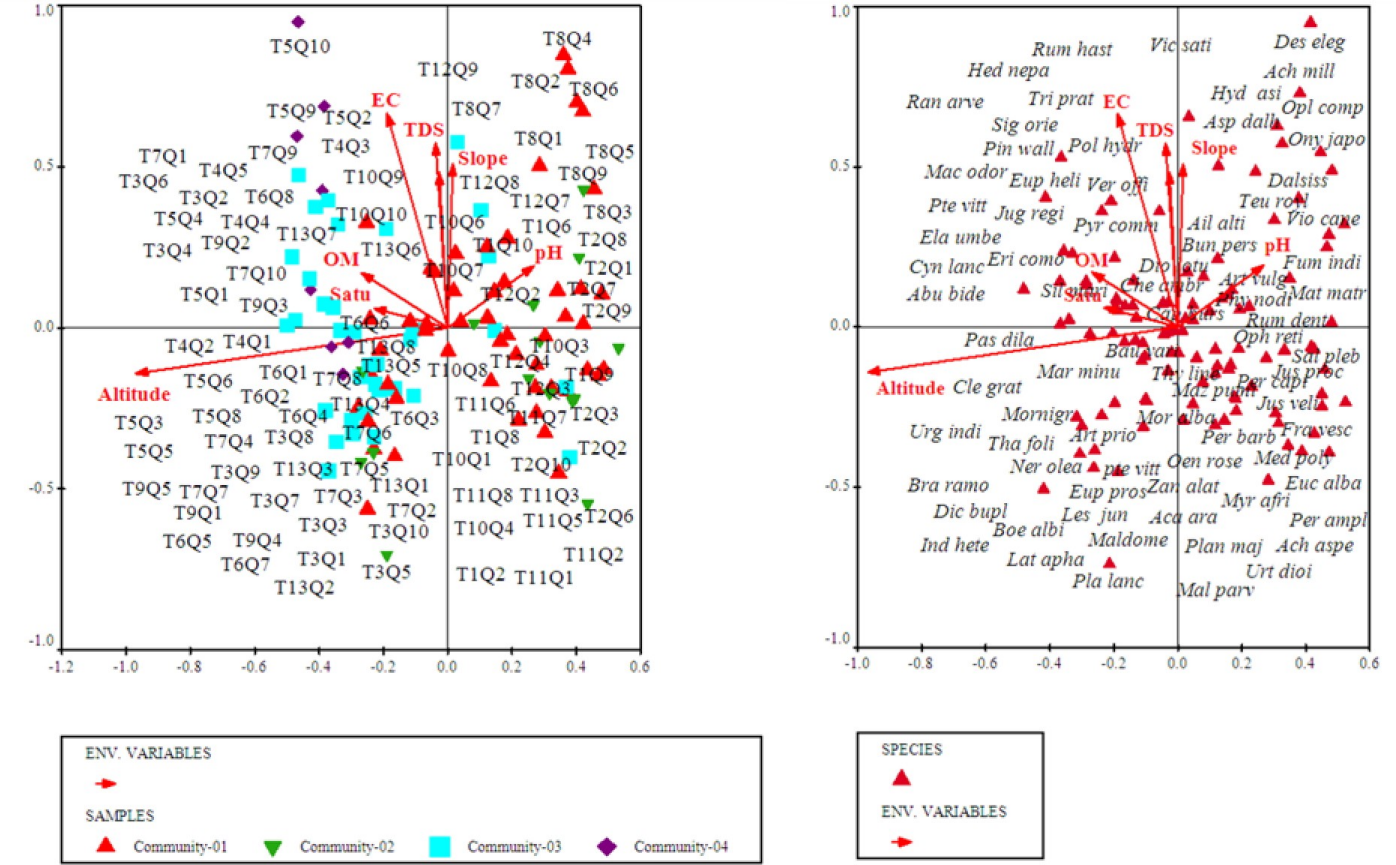
CCA biplots showing the distribution of plant species in relation to measured environmental factors.

Canonical Correspondence Analyses (CCA) using CANOCO software was applied to understand about the complex relationship among plant species composition and distribution in relation to measured environmental variables. The CCA (bi-plot diagram) of the first quadrant showed that most of the plant species were clustered around electrical conductivity, total dissolved solids, organic matter and saturation (Fig 7). While going through the second quadrant all the plant species were assembled under the influence of slope and soil pH. The 3^rd^ quadrant most of the plants clustered under the influence of altitude (Fig 7 and Table 1).

**Table 1:** Detailed summary CCA for all the recorded plant species and environmental factors.

**Axes**	**1**	**2**	**3**	**4**	**Total inertia**
Eigenvalues	0.433	0.259	0.214	0.183	17.982
Species-environment correlations	0.892	0.808	0.816	0.785	
Cumulative % variance of species data	2.4	3.8	5	6.1	
Cumulative % variance of species-environment relation	25.2	40.2	52.6	63.2	
Summary of Monte Carlo Test					
**Test of significance of first canonical axis**		**Test of significance of all canonical axes**			
eigen value	0.433	Trace		1.721	
F-ratio	2.592	F-ratio		1.389	
P-value	0.004	P-value		0.002	

## Discussion

In the present study, 62 plant families were reported in the Dhirkot region, District Bagh, Azad Jammu and Kashmir. Asteraceae and Lamiaceae were the leading families, followed by Rosaceae in the region [41]. Similarly, Nasir et al. [42] also reported these families as the most dominant in the Flora of Pakistan due to their unique characteristics and geographical properties. An adjacent location Amjad et al. [43] reported Asteraceae as the leading family, followed by Lamiaceae at District Kotli Azad Jammu and Kashmir. Similar to our work Khan et al. [33] also investigated and recorded the species density, cover and frequency of Mount Eelum Pakistan and reported 124 plant species distributed in 52 different families. The larger ecological amplitude and same environmental conditions are the reasons behind the dominancy of Asteraceae and Lamiaceae families in the region.

The four plant communities were identified after CA using PCORD software. Similar statistical approaches were also used by [35,44–48] for identification and classification of plant communities with similar floristic composition under the impact of different environmental variables. First community of the current study is the largest of all communities consisted of 54 different quadrats located at an altitude range of 968m to 1699m. The topmost indicator species of this community included *Olea ferruginea*, *Desmodium elegans* and *Prunella vulgaris*. The lower soil pH influence *Olea ferruginea* and *Desmodium elegans*. The 2^nd^ community is located from 972m to 1696m elevation with minimum number of stations. The topmost indicator species of this community included *Abies pindrow*, *Zanthoxylum alatum* and *Pteracanthus urticifolius*. High altitude influences *Abies pindrow* and low electrical conductivity influences the vegetation of *Pteracanthus urticifolius* species. The 3^rd^ community consisted of 36 different quadrats at an elevation range between 1097 to 1771 m. The topmost indicator species of this community included *Cedrus deodara*, *Elaeagnus umbellata* and *Hypericum perforatum*. The higher organic matter influence *Cedrus deodara* population in the region. The community 4 comprised of 44 different plant species at an elevation range between 1681 to 1738. The topmost indicator species of this community were *Alnus nitida*, *Myrsine africana* and *Ranunculus arvensis*. The low soil pH and saturation have significant effect on the indicators of this plant community. Similar to our study different researcher used the same approach for community classification and their respective indicators based on multivariate statistic approach like [26,44,49–52]. Our results are in great harmony with the findings of Khan et al. [44] where they recorded 5 plant communities, using PCORD software in Thandiani sub forest division of Western Himalayas. Where soil pH, mountain slope, aspect, soil electrical conductivity was the major environmental variables showing a significant effect on species composition. Whereas, same indicator species *Abies pindrow*, *Cedrus deodara* and *Zanthoxylum alatum* were also been reported. Rahman et al. [53] studied the plant communities of Peochar Valley of Hindu Kash Mountain and reported 4 plant communities based on Two-way Cluster Analysis where aspect, elevation, soil depth, grazing pressure and rock type were critical environmental factors affecting community composition pattern.

Furthermore, the measured edaphic factors have a significant role in vegetation of the region. Similar to our finding Ali et al. [54] also reported that the vegetation and soil of a specific region have a strong relationship with each other. The soil is an essential factor that plays a crucial role in plant selection through evolutionary change [55]. Like to our current study different researchers have observed effects of soil pH, aspect and slope on different species in other mountainous regions [56–59]. The optimum soil pH for the availability of nutrients is between 5 and 7.5 and the greatest availability at 6.5 [60]. The soil pH of the Dhirkot region is basic that was in close harmony with the findings of Amjad et al. [43], in the Nikyal Valley. In the current study, high organic matter was observed that also significantly influences plant species composition and distribution pattern. The colloidal nature and the soil’s water-investment ability were subsequently increased in the number of plant communities with more significant soil organic matter [61].

In the current study, CCA was carried out for ordination analysis. It is mainly used as analytical techniques in ecological studies to determine discrete units, ecological gradients, and a significant relationship between environmental and floristic data [16,62,63]. In the current research project, CCA revealed the significant impact (p<0.05) of measured environmental factors on 145 plant species of 4 plant communities. These statistical techniques were also used by Janbeen and Ahmad [64] to evaluate the relationship between vegetation and soil at Ayub National Park in Pakistan. Furthermore, multivariate techniques were also used by Ahmad et al. [65] to assess the ecological aspect of vegetation around Havalian city. Identification of indicator species in the current study is a novel approach for prioritizing species of ecological importance of a region and can be used on broader scale for the vegetation of other regions of Hindu-Himalayas.

## Conclusion

It was concluded that multivariate statistic approach i.e., cluster analysis, indicator species and canonical correspondence analysis are one of the significant methods to classify the vegetation into different plant communities/association. In the current study soil pH, electrical conductivity, saturation, altitude, and organic matter concentration are factors responsible for the formation of plant communities’ formation and their respective indicators of the Dhirkot region, Azad Jammu and Kashmir, Pakistan. Based on our findings it’s recommended that the identified indicator and rare plant species of the investigated area can further be evaluated and utilized in the afforestation and reforestation drives by the relevant departments.

## Supporting information

S1 Table Detailed list of reported plant species along with their habit and family of the Dhirkot Valley, District Bagh Azad Kashmir, Pakistan. (DOCX)
