# Expression of concern: Phyto-ecological studies and distribution pattern of plant species and communities of Dhirkot, Azad Jammu and Kashmir, Pakistan

**DOI:** 10.1371/journal.pone.0351613

**Published:** 2026-06-15

**Authors:** The PLOS One Editors

The *PLOS One* Editors issue this Expression of Concern for this article [1] because of concerns identified about the peer review process. Readers are advised to interpret the article [1] with caution.
