# Supplementary material for: Phyto-ecological studies and distribution pattern of plant species and communities of Dhirkot, Azad Jammu and Kashmir, Pakistan
Source: PLoS One. 2021 Oct 6;16(10):e0257493. doi: 10.1371/journal.pone.0257493 (PMC8494300; doi:10.1371/journal.pone.0257493)
Supplement: S1 Table — (DOCX) [file pone.0257493.s001.docx]

**Supplementary Table 1. Detailed list of reported plant species along with their habit and family of the Dhirkot Valley, District Bagh Azad Kashmir, Pakistan.**

| **S.No** | **Plant Name** | **Habit** | **Family** |
| --- | --- | --- | --- |
| 1 | *Acacia arabica* | Tree | Leguminosae |
| 2 | *Aesculus indica*(Wall. ex Cambess.) Hook | Tree | Sapindaceae |
| 3 | *Bauhinia variegata*L | Tree | Caesalpiniaceae |
| 4 | *Citrus aurantium* L | Tree | Rutaceae |
| 5 | *Dalbergia sissoo*DC | Tree | Leguminosae |
| 6 | *Eucalyptus alba*Reinw. ex Blume | Tree | Myrtaceae |
| 7 | *Ficus palmata*Forssk | Tree | Moraceae |
| 8 | *Pyrus pseudopashia*T.T.Yu | Tree | Rosaceae |
| 9 | *Juglans nigra*L | Tree | Juglandaceae |
| 10 | *Malus domestica*Borkh | Tree | Rosaceae |
| 11 | *Melia azedarach*L. | Tree | Meliaceae |
| 12 | *Morus nigra*L | Tree | Moraceae |
| 13 | *Morus alba*L | Tree | Moraceae |
| 14 | *Olea ferruginea* Wall. ex Aitch*.* | Tree | Oleaceae |
| 15 | *Pinus roxburghii*Sarg | Tree | Pinaceae |
| 16 | *Prunus domestica*L | Tree | Rosaceae |
| 17 | *Prunus armeniaca*L | Tree | Rosaceae |
| 18 | *Prunus persica*(L.) Batsch | Tree | Rosaceae |
| 19 | *Pyrus communis*L | Tree | Rosaceae |
| 20 | *Quercus incana*Bartram | Tree | Fagraceae |
| 21 | *Pinus wallichiana*A.B.Jacks | Tree | Pinaceae |
| 22 | *Diospyros lotus*L | Tree | Ebenaceae |
| 23 | *Ailanthus altissima*(Mill.) Swingle | Tree | Simaroubaceae |
| 24 | *Salix nigra*Marshall | Tree | Salicaceae |
| 25 | *Abies pindrow*(Royle ex D.Don) Royle | Tree | Pinaceae |
| 26 | *Ficus carica*L | Tree | Moraceae |
| 27 | *Platanus orientalis*L | Tree | Platanaceae |
| 28 | *Sapindus mukorossi*Gaertn | Tree | Sapindaceae |
| 29 | *Cedrus deodara*(Roxb. ex D.Don) G.Don | Tree | Pinaceae |
| 30 | *Alnus nitida*(Spach) Endl | Tree | Betulaceae |
| 31 | *Indigofera heterantha*Brandis | Shrub | Leguminosae |
| 32 | *Elaeagnus umbellata*Thunb | Shrub | Elaeagnaceae |
| 33 | *Punica granatum*L | Shrub | Lythraceae |
| 34 | *Viburnum grandiflorum*Wall. ex DC | Shrub | Adoxaceae |
| 35 | *Machilus odoratissim* | Shrub | Lauraceae |
| 36 | *Berberis lycium*Royle | Shrub | Berberidaceae |
| 37 | *Rosa bronneri* | Shrub | Rosaceae |
| 38 | *Myrsine africana*L | Shrub | Primulaceae |
| 39 | *Dodonaea viscosa*(L.) Jacq | Shrub | Sapindaceae |
| 40 | *Sarcococca saligna* | Shrub | Buxaceae |
| 41 | *Rubus ellipticus*Sm | Shrub | Rosaceae |
| 42 | *Isodon rugosus*(Wall. ex Benth.) Codd | Shrub | Lamiaceae |
| 43 | *Zanthoxylum alatum* | Shrub | Rutaceae |
| 44 | *Nerium oleander*L | Shrub | Apocynaceae |
| 45 | *Wikstroemia canescens*Wall. ex Meisn | Shrub | Thymelaeaceae |
| 46 | *Debregeasia salicifolia* | Shrub | Urticaceae |
| 47 | *Jasminum grandiflorum*L | Shrub | Oleaceae |
| 48 | *Ricinus communis*L | Shrub | Euphorbiaceae |
| 49 | *Desmodium elegans*DC | Shrub | Leguminosae |
| 50 | *Avena fatua*L | Herb | Poaceae |
| 51 | *Euphorbia helioscopia*L | Herb | Euphorbiaceae |
| 52 | *Hedera nepalensis*K.Koch | Herb | Araliaceae |
| 53 | *Amaranthus spinosus*L | Herb | Amaranthaceae |
| 54 | *Mazus pumilus*(Burm.f.) Steenis | Herb | Mazaceae |
| 55 | *Traxicum officinalis* | Herb | Asteraceae |
| 56 | *Gerbera gossypina*(Royle) Beauverd | Herb | Asteraceae |
| 57 | *Filago hurdwarica*(Wall. ex DC.) Wagenitz | Herb | Compositae |
| 58 | *Ranunculus arvensis*L | Herb | Ranunculaceae |
| 59 | *Dryopteris filix-mas*(L.) Schott | Herb | Dryopteridaceae |
| 60 | *Silybum marianum*(L.) Gaertn | Herb | Compositae |
| 61 | *Rumex hastatus*D. Don | Herb | Polygonaceae |
| 62 | *Eriophorum comosum*(Wall.) Nees | Herb | Cyperaceae |
| 63 | *Abutilon bidentatum*Hochst. ex A.Rich | Herb | Malvaceae |
| 64 | *Clematis grata* Wall./AF-78 | Herb | Ranunculaceae |
| 65 | *Rumex dentatus*L | Herb | Polygonaceae |
| 66 | *Impatiens edgeworthii*Hook. F | Herb | Balsaminaceae |
| 67 | *Cynoglossum lanceolatum*Forssk | Herb | Boraginaceae |
| 68 | *Fragaria nubicola*(Lindl. ex Hook.f.) Lacaita | Herb | Rosaceae |
| 69 | *Arthraxon prionodes*(Steud.) Dandy | Herb | Poaceae |
| 70 | *Achyranthes aspera*L | Herb | Amaranthaceae |
| 71 | *Malvastrum coromandelianum*(L.) Garcke | Herb | Malvaceae |
| 72 | *Oenothera rosea*L'Hér. ex Aiton | Herb | Onagraceae |
| 73 | *Cannabis sativa*L | Herb | Cannabacea |
| 74 | *Mentha longifolia (*L.) L | Herb | Lmiaceae |
| 75 | *Ipomoea purpurea(*L.)Roth./AF-76 | Herb | Convolvulaceae |
| 76 | *Polygonium amplexicaulis* | Herb | Polygonaceae |
| 77 | *Onychium japonicum*var. lucidum (D. Don) Christ | Herb | Pteridaceae |
| 78 | *Medicago polymorpha*L. | Herb | Fabaceae |
| 79 | *Micromeria biflora*(Buch.-Ham. ex D.Don)Benth | Herb | Lamiacea |
| 80 | *Urtica dioica*L | Herb | Urticaceae |
| 81 | *Tagetes minuta*L | Herb | Asteraceae |
| 82 | *Pteris cretica*L | Herb | Poaceae |
| 83 | *Vicia sativa*L | Herb | Leguminosae |
| 84 | *Parthenium hysterophorus*L | Herb | Asteraceae |
| 85 | *Plantago lanceolata*L | Herb | Plantaginaceae |
| 86 | *Phyla nodiflora*(L.) Greene | Herb | Verbenaceae |
| 87 | *Achillea millefolium*L. | Herb | Asteraceae |
| 88 | *Hydrocotyl asiatica* | Herb | Araliaceae |
| 89 | *Mentha arvensis*L | Herb | Lamiaceae |
| 90 | *Sigesbeckia orientalis*L | Herb | Asteraceae |
| 91 | *Plantago major*L | Herb | Plantaginaceae |
| 92 | *Solanum nigrum*L | Herb | Solanaceae |
| 93 | *Boenninghausenia albiflora*(Hook.) Rchb. ex Meisn | Herb | Rutaceae |
| 94 | *Lonicera webbiana*Wall. ex DC | Herb | Caprifoliaceae |
| 95 | *Salvia plebeia*R.Br | Herb | Lamiaceae |
| 96 | *Matricaria matricarioides*(Less.) Porter | Herb | Asteraceae |
| 97 | *Justicia procumbens*L | Herb | Acanthaceae |
| 98 | *Persicaria barbata*(L.) H.Hara | Herb | [Polygonaceae](http://www.theplantlist.org/1.1/browse/A/Polygonaceae/) |
| 99 | *Scutellaria chamaedrifolia*Hedge &A.J.Paton | Herb | Lamiaceae |
| 100 | *Ophioglossum reticulatum*L | Herb | Ophioglossaceae |
| 101 | *Ajuga parviflora*Benth | Herb | Lamiacea |
| 102 | *Euphorbia indica*Lam/AF-15 | Herb | Euphorbiaceae |
| 103 | *Cichorium intybus*L | Herb | Asteraceae |
| 104 | *Cymbopogon martini*(Roxb.) W.Watson | Herb | Poaceae |
| 105 | *Swertia paniculata*Wall | Herb | Gentianaceae |
| 106 | *Bromus catharticus*Vahl | Herb | Poaceae |
| 107 | *Polygonium hydropiper* | Herb | Polygonaceae |
| 108 | *Ranunculus muricatus*L | Herb | Ranunculaceae |
| 109 | *Fragaria vesca*L | Herb | Rosaceae |
| 110 | *Marsilea minuta*L | Herb | Marsileaceae |
| 111 | *Persicaria amplexicaulis*(D.Don) RonseDecr | Herb | Polygonaceae |
| 112 | *Asplenium dalhousiae*Hook | Herb | Aspleniaceae |
| 113 | *Pteracanthus urticifolius*(Wall. ex Kuntze) Bremek | Herb | Acanthaceae |
| 114 | *Teucrium royleanum*Wall. ex Benth | Herb | Lamiaceae |
| 115 | *Lathyrus aphaca*L | Herb | Leguminosae |
| 116 | *Lespedeza juncea*(L.f.) Pers | Herb | Leguminosae |
| 117 | *Verbena officinalis*L | Herb | Verbenaceae |
| 118 | *Pteris vittata*L | Herb | Pteridaceae |
| 119 | *Brachiaria ramosa*(L.) Stapf | Herb | Poaceae |
| 120 | *Euphorbia prostrata*Aiton | Herb | Euphorbiaceae |
| 121 | *Dicliptera bupleuroides*Nees | Herb | Acanthaceae |
| 122 | *Trifolium pratense*L. | Herb | Leguminosae |
| 123 | *Oplismenus compositus*(L.) P.Beauv. | Herb | Poaceae |
| 124 | *Malva parviflora*L. i | Herb | Malvaceae |
| 125 | *Thymus linearis*Benth. | Herb | Lamiaceae |
| 126 | *Bunium persicum*(Boiss.) B.Fedtsch | Herb | Apiaceae |
| 127 | *Fumaria indica*(Hausskn.) Pugsley | Herb | Papaveraceae |
| 128 | *Oxalis corniculata*L | Herb | Oxalidaceae |
| 129 | *Artemisia vulgaris*L | Herb | Asteraceae |
| 130 | *Chenopodium ambrosoide* | Herb | Amaranthaceae |
| 131 | *Ajuga bracteosa* | Herb | Lamiacea |
| 132 | *Anaphalis barnesii*C.E.C.Fisch | Herb | Compositae |
| 133 | *Paspalum dilatatum*Poir | Herb | Polygonaceae |
| 134 | *Capsella bursa-pastoris*(L.) Medik | Herb | Brassicaceae |
| 135 | *Urginea indica* | Herb | Liliaceae |
| 136 | *Scutellaria petiolata*Hemsl. ex Lace &Prain | Herb | Lamiaceae |
| 137 | *Persicaria capitata*(Buch.-Ham. ex D.Don) H.Gros | Herb | Polygonaceae |
| 138 | *Justicia velizii*T.F. Daniel | Herb | Acanthaceae |
| 139 | *Swertia cordata*(Wall. ex G. Don) C.B. Clark | Herb | Gentianaceae |
| 140 | *Campanula pallida*Wall. | Herb | Campanulaceae |
| 141 | *Thalictrum foliolosum*DC | Herb | Ranunculaceae |
| 142 | *Hypericum perfoliatum*L | Herb | Hypericaceae |
| 143 | *Clematis graveolens*Lindl | Herb | Ranunculaceae |
| 144 | *Viola canescens*Wall. | Herb | Violaceae |
| 145 | *Prunella vulgaris*L | Herb | Lamiacea |
